# The Use of Aluminosilicate Ash Microspheres from Waste Ash and Slag Mixtures in Gypsum-Lime Compositions

**DOI:** 10.3390/ma16124213

**Published:** 2023-06-06

**Authors:** Victoria Petropavlovskaya, Maria Zavadko, Tatiana Novichenkova, Kirill Petropavlovskii, Mikhail Sulman

**Affiliations:** Faculty of Civil Engineering, Tver State Technical University, 170026 Tver, Russia

**Keywords:** gypsum, fly ash, aluminosilicate microsphere, slaked lime, water demand, repair composition

## Abstract

The article considered the issues of the modification of gypsum stone to improve its performance properties. The influence of mineral additives on the physical and mechanical characteristics of the modified gypsum composition is described. The composition of the gypsum mixture included slaked lime and an aluminosilicate additive in the form of ash microspheres. It was isolated from ash and slag waste from fuel power plants as a result of their enrichment. This made it possible to reduce the carbon content in the additive to 3%. Modified compositions of the gypsum composition are proposed. The binder was replaced with an aluminosilicate microsphere. Hydrated lime was used to activate it. Its content varied: 0, 2, 4, 6, 8 and 10% of the weight of the gypsum binder. Replacing the binder with an aluminosilicate product for the enrichment of ash and slag mixtures made it possible to improve the structure of the stone and increase its operational properties. The compressive strength of the gypsum stone was 9 MPa. This is more than 100% higher than the strength of the control composition of gypsum stone. Studies have confirmed the effectiveness of using an aluminosilicate additive—a product of enrichment of ash and slag mixtures. The use of an aluminosilicate component for the production of modified gypsum mixtures allows the saving of gypsum resources. Developed formulations of gypsum compositions using aluminosilicate microspheres and chemical additives provide the specified performance properties. This makes it possible to use them in the production of self-leveling floors, plastering and puttying works. Replacing traditional compositions with a new composition based on waste has a positive effect on the preservation of the natural environment and contributes to the formation of comfortable conditions for human habitation.

## 1. Introduction

Low risks in the energy supply of coal-fired power plants are associated with the high availability of coal. It causes the widespread distribution of thermal power plants that run on coal fuel. The prevalence of coal-fired power plants persists against the backdrop of a shift in the interests of many states towards renewable energy.

When burning solid fuel, a large amount of ash and slag waste is generated, the storage of which in ash dumps is a source of soil and groundwater pollution [1,2,3] and leads to an annually increasing level of environmental pollution threat. In this regard, the issues of the most efficient use of stored ash and slag waste as secondary resources are relevant today [4].

Ash and slag waste can be classified into 3 groups:

- fly ash (dry removal, sedimentation of ash particles in cyclones and electro-filters, accumulation in silos)
- fuel slags (melt precipitation at complete melting of part of the fuel in the lower part of the boiler furnace)
- ash and slag mixture (wet removal, directed to the ash dump in the form of pulp).

Fly ash is the most used in secondary production today. Pozzolanic activity of such ash is due to the presence of amorphous aluminosilicate. This allows it to be used as a replacement for part of the cement in the production of concrete. It has been proven that the use of ash as an additive in concrete makes it possible to reduce the use of cement without compromising durability [2,5].

The greatest number of difficulties are associated with hydro-removal ash in terms of the development of a technology for recycling it into the production of new materials. These are due to the heterogeneity of its composition, properties and its contamination. This makes it practically impossible to use hydro-removal dump ash without preliminary preparation and separation into its components by the flotation method.

The aluminosilicate component has the greatest value from the point of view of practical application. The aluminosilicate component is composed of spheroidal nano- and micro-sized particles with a shell of pure alumina, a free-flowing product that can be used as fillers in building composites to reduce their cost, increase wear resistance and insulating properties, as well as increase physical and technical characteristics by obtaining the optimal packing density and the maximum number of particle contacts.

The use of an optimal amount of aluminosilicate microspheres in the composition of foam concrete, corresponding to 5–10% by weight of cement, makes it possible to increase the strength of the stone on the 28th day of hardening by an average of 40% [6].

The use of ash components on the nanoscale level significantly increases the efficiency of their use in the composition of concrete and allows the greatest increase in the strength of artificial stone [7]. Compositions containing ash components of a fine fraction make it possible, among other things, to solve particular problems in the field of reconstruction [8]. There are also possible ways to increase the efficiency of the use of ash waste through their use not only as a filler, but also as an active mineral additive [9]. Known methods of using include the ash of agricultural waste in concrete as a pozzolana [10]. Many authors note that ash as a pozzolan in concrete shows the same effect as a natural pozzolan, which indicates its great potential for use. It was shown in [11] that the use of ash contributes to the improvement of characteristics in terms of mechanical properties, especially at later stages of artificial stone hardening. Researchers also use natural pozzolans and ash together in the composition, which in turn achieves the best concrete characteristics [12].

As a rule, researchers consider the use of ash and slag waste components in systems based on a cement binder [13,14]. But today, due to the need to develop directions for alternative types of binders, due to an increase in anthropogenic gas emissions by the cement industry [15], there is an interest in modifying cementless or composite systems (when cement is not the only type of binder, which allows the reduction of its consumption) [16]. In [17], it is noted that in the future, the growth of cement production will continue, which requires the development of optimized mixture compositions, in terms of cement consumption. The scale of cement production leads to more than 7% of annual anthropogenic greenhouse gas emissions arising from both energy use and chemical reactions [18]. In addition, the cement industry harms the environment not only through carbon dioxide emissions, but also cement dust emissions that affect soil quality [19]. Thus, the use of ashes in the form of components of binders, both cement and alternative, is an important task to prevent their harmful effects on the environment [20,21].

Composite gypsum compositions may be of interest due to the great possibilities for their characteristics involved with the modification and increase in strength. For example, the formation of calcium hydrosulfoaluminates after the appearance of the primary crystalline framework of sulfate dihydrate with the introduction of active mineral additives is one of the ways to influence the strength characteristics of a stone on a gypsum binder [22].

Research aimed at obtaining gypsum compositions is especially relevant today, including with the development of 3D technologies [23]. Studies [24] provide a detailed discussion of the use of alternative binders specifically for 3D printing, where special attention is paid to gypsum binders. Of particular interest are the results of studies on the development of mixture compositions based on a gypsum-cement-pozzolanic binder for 3D printing [25,26].

There are several ways to increase the strength of gypsum stone, the most common today is the method of using finely dispersed mineral fillers [27]. To a lesser extent, the method of influence by introducing additives on the morphology of the resulting crys-talline hydrates in the structure of the modified gypsum matrix is known [28]. One of the ways to increase the strength of gypsum stone is also via the introduction of nanocomponents into the composition [29], however, this method remains the most expensive to date [30]. Obtaining effective gypsum compositions is possible through a combination of the above methods. For example, rational compositions of gypsum compositions for various purposes were obtained by the authors of [31] using several approaches to their design.

The use of waste as modifying additives in the composition of gypsum compositions makes it possible to simultaneously achieve a significant economic effect [32,33]. In [32], the effect of added plastic polycarbonate waste on the mechanical and environmental properties of composites with a gypsum matrix was analyzed: the conducted studies proved the effectiveness of this approach. In the case of gypsum compositions, the use of waste is possible not only as modifying additives, but also as the main component [34,35].

Thus, the use of small aggregates in the form of ash and slag waste components has a positive effect on the strength characteristics of artificial stone on alternative types of binder [36,37,38,39], i.e., its use is possible not only in systems based on a cement binder, as noted in [40], and concrete [41,42]. As a rule, a positive effect is due not only to packing density [43], but also to a change in the morphology of gypsum crystals formed during hydration and curing [43]. Thus, several approaches to the design of the structure were used at once [31], the introduction of an aluminosilicate component also plays the role of a filler [27] and affects the microstructure by changing the morphology and the formation of neoplasms [28].

According to the analysis of sources, to date, ash and slag wastes are used mainly in compositions based on a cement binder, thus, the novelty of this work lies in the proposed effective method for using such components in cementless compositions. The purpose of these studies was to establish the regularities of the influence of aluminosilicate microspheres obtained by enriching the ash and slag mixture on the processes of structure formation of gypsum-lime compositions and properties. 

## 2. Materials and Methods

**Figure 1:**
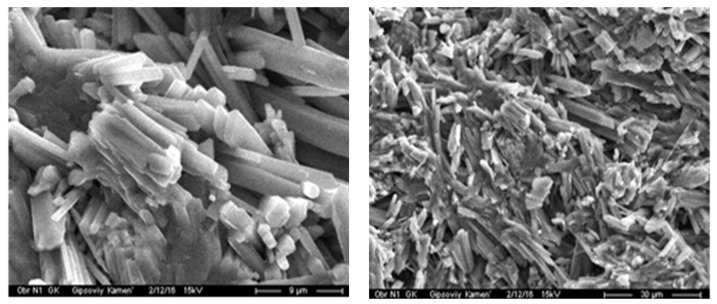
The gypsum structure of the control composition.

When conducting experimental studies, a gypsum binder of a β-modification (Samara region) was used as a starting material. Characteristics of a low-temperature calcined gypsum binder: compressive strength—4 MPa, flexural strength—2 MPa, residue on a sieve of 0.2 mm—of no more than 1%. The microstructure of the original gypsum binder is shown in Figure 1.

The composition also used slaked lime (Novgorod region). The quality indicators of lime corresponded to grade 1 according to the requirements of GOST 9179-2018 [44] Lime for building purposes. Specifications. The content of active CaO + MgO − 97.3%.

**Figure 2:**
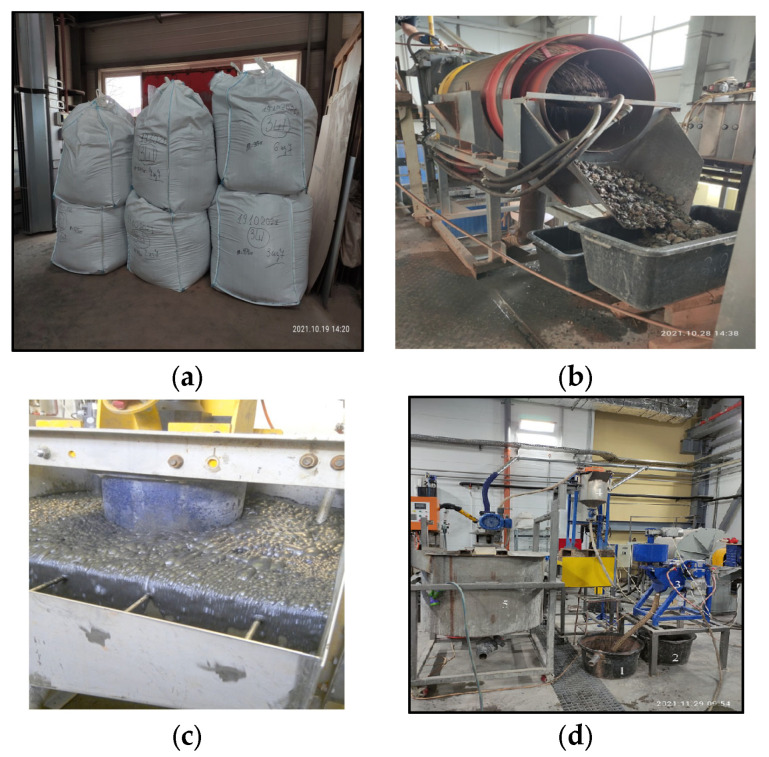
Obtaining aluminosilicate microspheres by the enrichment of ash and slag waste: (**a**) ash and slag waste, (**b**) dry classification, (**c**) main flotation, (**d**) magnetic separation.

**Figure 3:**
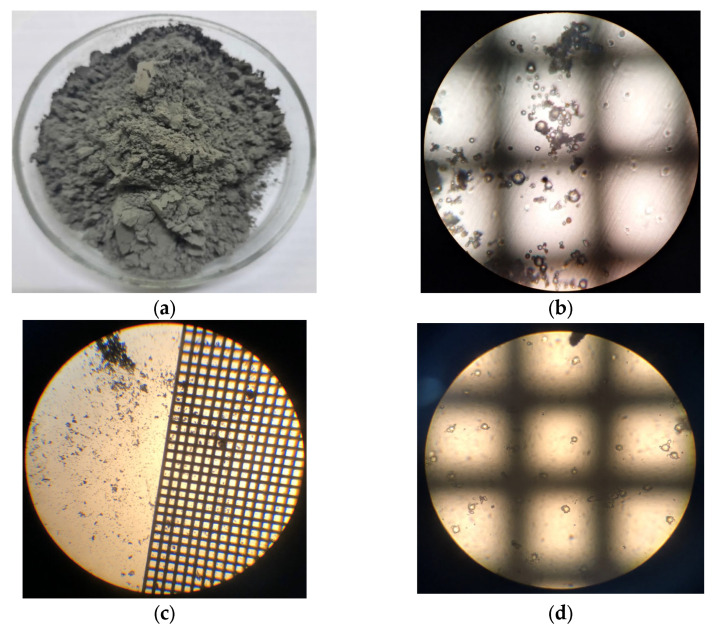
Appearance of the aluminosilicate component.

The starting material for obtaining aluminosilicate ash microspheres was ash and slag waste (Figure 2a) from fuel power plants in the Moscow region. The process of enrichment of the ash and slag mixture included the following stages: dry classification (Figure 2b), main flotation (Figure 2c), control flotation, magnetic separation (Figure 2d) and screening [38]. Magnetic separation after flotation made it possible to remove magnetically susceptible inclusions by the pulp flow, which, under the influence of a magnetic field, were attracted to the magnetic system of the drum and moved by its rotating shell to the discharge zone. Further, under the action of the pressure of water supplied through the nozzles located along the magnetic drum, the magnetic particles were discharged into the unloading chute, and the pure aluminosilicate component passed the last stage—screening. Such a technological scheme made it possible to separate the ash and slag mixture into components and ensure the constancy of the composition and properties of each of the components, including aluminosilicate (Figure 3a). As a component in the compositions, an enriched product of ash and slag waste processing, an aluminosilicate ash microsphere (Figure 3b), was used.

**Figure 4:**
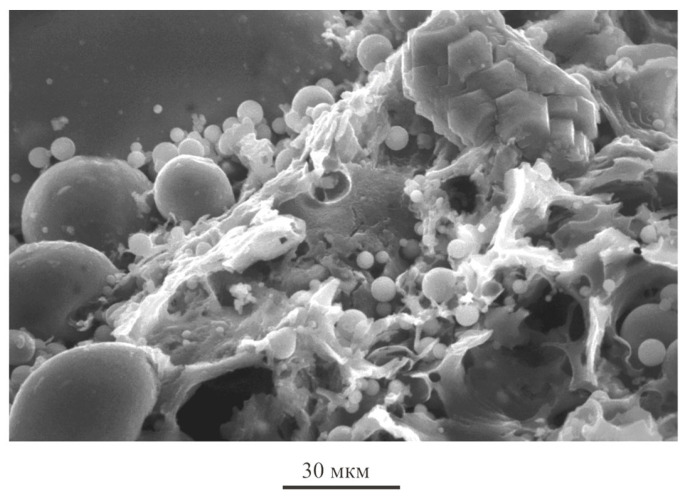
Morphological features of aluminosilicate microspheres.

A high-quality aluminosilicate component was obtained (Figure 3c). It met the highest requirements for additives in the construction industry. The chemical composition of the aluminosilicate microsphere is presented in Table 1. It contained about 3.0% carbon. The structure of the aluminosilicate component (Figure 3c) is represented by an amorphous phase (Figure 3d). In some areas of the aluminosilicate enrichment product, crystalline formations of quartz are observed (Figure 4).

**Figure 5:**
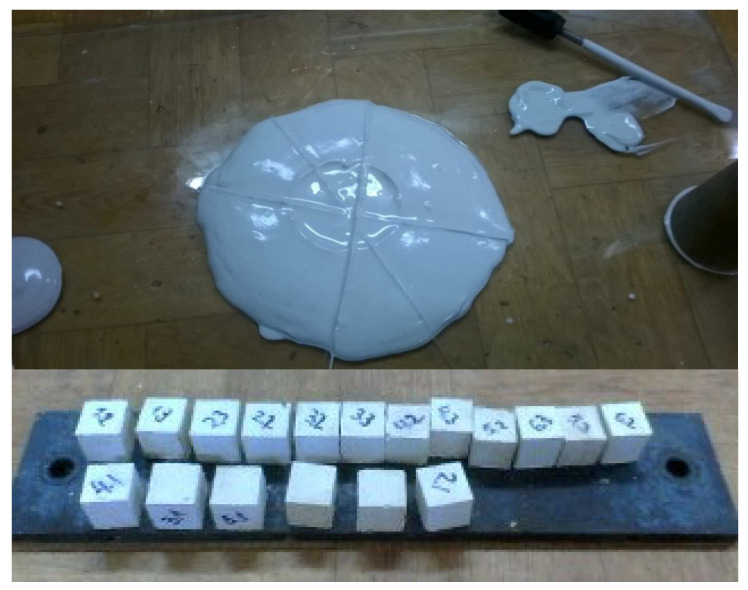
Determination of the properties of the low-temperature calcined gypsum binder.

**Figure 6:**
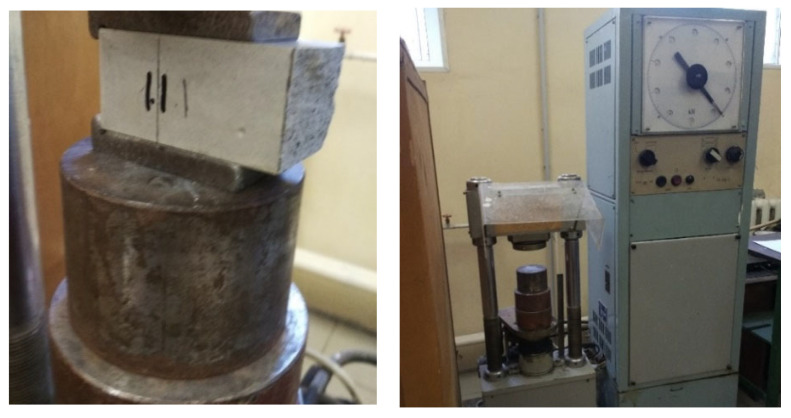
Tests of sample-beams of modified gypsum.

The physical and mechanical properties of the raw materials (Figure 5), as well as the resulting composites (Figure 6), were determined in an accredited laboratory of the Tver State Technical University. The manufacture of standard samples with dimensions of 40 × 40 × 160 mm and samples-cubes with dimensions of 20 × 20 × 20 mm was carried out in accordance with GOST 23789-2018 [45] «Gypsum binders. Test methods». Studies of the main physical and mechanical properties of the developed compositions were carried out in accordance with: GOST R 58766-2019 [46] «Construction mortars. General technical conditions», GOST 23789-2018 «Gypsum binders. Test methods», GOST 31358-2019 [47] «Dry construction floor mixes», GOST 31376-2008 [48] «Dry construction mixes based on gypsum binder». Electron microscopic studies of the microstructure were carried out on a JEOL JSM-6610LV SEM scanning electron microscope («Jeol», Japan). To determine the topography of the sample, the mode of secondary electrons was used, and to study the phase and chemical composition of the sample, the mode of reflected (back-scattered) electrons was used. Elemental chemical analysis of the samples was carried out using an analytical attachment to a scanning electron microscope of an Oxford INCA Energy 350 X-ray energy-dispersive microanalysis system («Oxford Instruments Analytical», Great Britain).

The molding of the samples was carried out by casting a gypsum mixture of a standard consistency also in accordance with GOST 23789-2018. Dough with a melt diameter (180 ± 5) mm was taken as a standard consistency. Hardening under normal conditions was carried out in a normal hardening chamber at a temperature of (+20 ± 2) °C and a relative air humidity of 75% for 7 days.

## 3. Research Results

### 3.1. The Mineral Composition of the Composition

**Figure 7:**
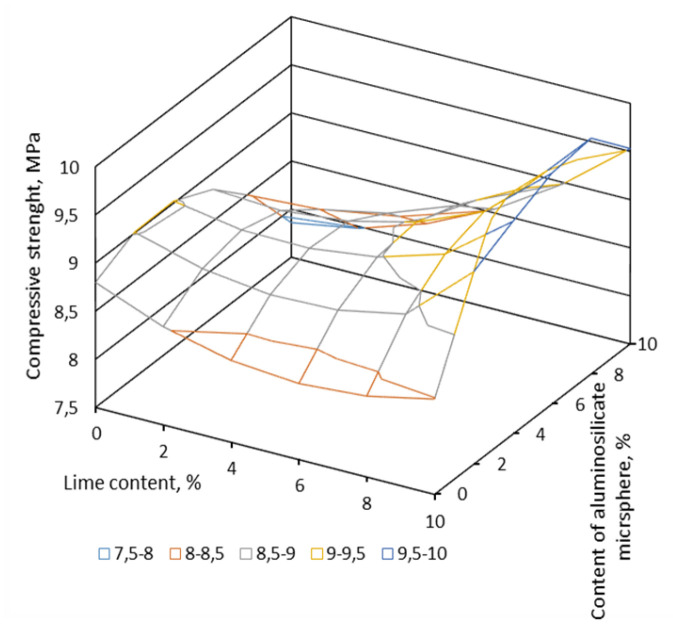
Influence of the content of aluminosilicate microspheres and slaked lime on compressive strength.

**Figure 8:**
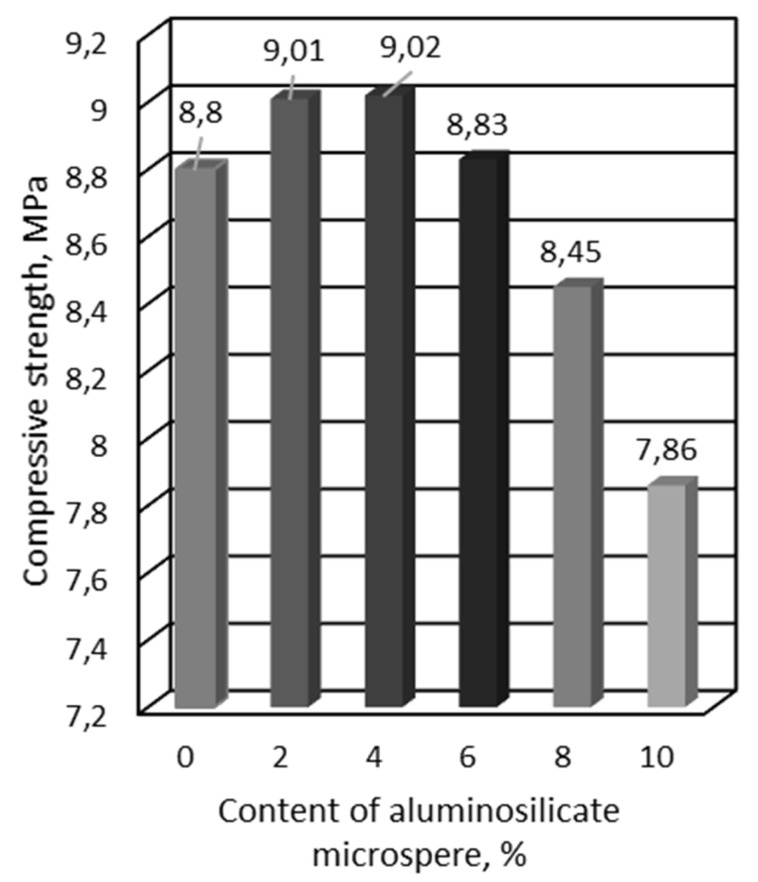
Influence of aluminosilicate microspheres on the compressive strength at a constant content of slaked lime in the amount of 10%.

**Figure 9:**
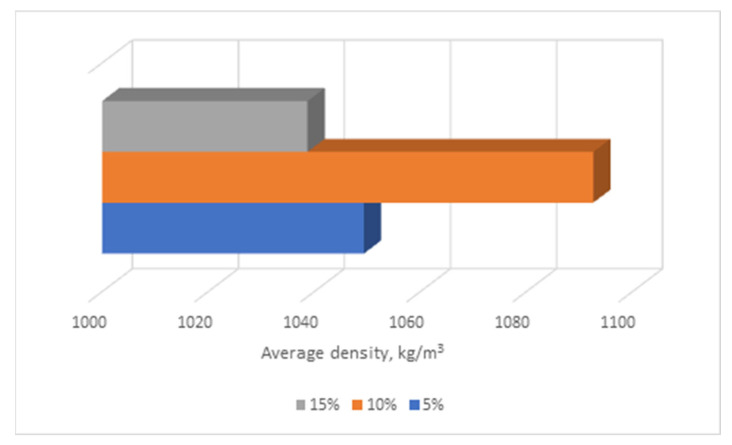
Effect of lime content on the average density of modified gypsum.

At the first stage of research, the possibility of using an aluminosilicate microsphere as part of a composite gypsum-lime binder was studied. For research, mathematical planning of the experiment was applied. Two factors were chosen. They determined the content of the aluminosilicate microspheres in percent (X1) and the content of slaked lime in percent (X2). As a result of the experiment, the dependences of the compressive strength on the content of these components were obtained (Figure 7, Figure 8 and Figure 9).

According to the results of the planned experiment, the optimal content of the components was «gypsum binder»:«hydrated lime»:«aluminosilicate microsphere» as 17:1.7:1, respectively. Hydrated lime in the pore liquid CaSO_4_ 2H_2_O activates the vitreous phase of the aluminosilicate microsphere. This contributes to the early release of ettringite and increases the strength of the stone. Studies of the mineralogical composition of samples of optimal composition showed the content of ettringite in the amount of 13.4% in those samples that were modified with aluminosilicate microsphere and slaked lime (Table 2). The microstructure of the sample of optimal composition is shown in Figure 6.

**Figure 10:**
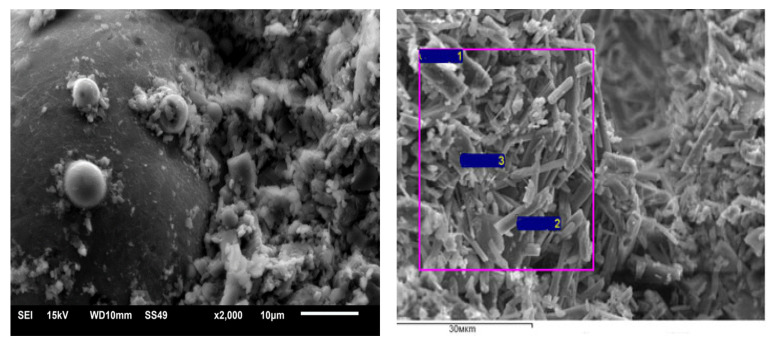
Micrographs of modified gypsum stone.

**Figure 11:**
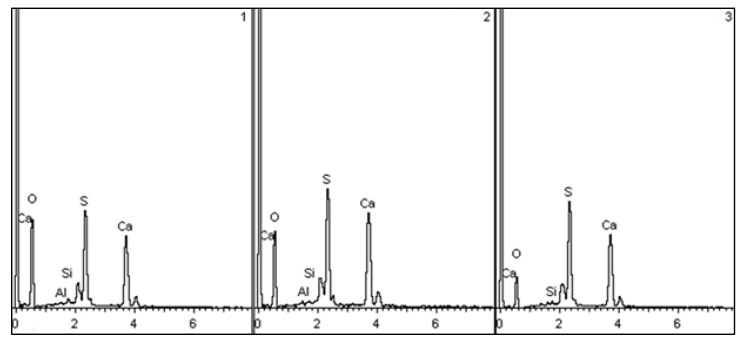
Registered spectra of modified gypsum stone.

Microstructural (Figure 10) and spectral analysis of the control gypsum stone and gypsum-lime stone (Figure 11 and Table 3) showed the active crystallization of gypsum on the surface of the aluminosilicate microsphere with the subsequent formation of a high-strength fine-crystalline structure of the stone.

On the spectrograms, there is an overlap of the reflections of a crystallizing substance—submicroparticles of calcium sulfate dihydrate and a «substrate». Aluminosilicate microspheres act as crystallization centers—«substrates». 

### 3.2. Optimization of the Prescription Composition of the Compositions

At the second stage, the formulations of the obtained compositions with chemical additives were studied. The introduction of plasticizing, water-retaining, and defoaming agents, shrinkage compensators and a setting time regulator into the formulations was due to a set of requirements for the technological and operational properties of gypsum compositions. Complexes of additives in the indicated concentrations (Table 4) provide the necessary characteristics for raw mixtures. Such formulations can be used for the manufacture of dry building mixes. The water-retaining additive (water-soluble cellulose ether and methyl-hydroxypropyl cellulose) and the polycarboxylate superplasticizer provide high mobility and good spreadability of the mortar mixture. Tartaric acid allows you to get the optimal pot life of the solution. The powder defoamer promotes the formation of an aesthetically homogeneous surface without air bubbles during curing. The shrinkage compensator makes it possible to avoid possible deformations during the hardening of the modified gypsum stone in cases where the formulations of the mixtures are offered for the manufacture of self-leveling floors. The optimal ratios of all the components of raw mixtures were established (Table 4). 

## 4. Discussion

Experimental studies have established that the aluminosilicate product of the enrichment of ash and slag mixtures is an active mineral additive in compositions based on a gypsum-lime binder [27]. The increase in the strength of the modified gypsum stone with the joint introduction of aluminosilicate microspheres and slaked lime is due to the formation of a finely crystalline structure of the composition, which was also noted by the authors of the work [28]. The multi-directionality of crystals in the structure of the modified stone is noted. Such a structure has enhanced performance characteristics. In the presence of a microdispersed additive, additional structural bonds are formed together with slaked lime. There is an additional compaction of the structure of the modified artificial stone. This is also facilitated by the mineralogical presence in the composition of the ash product—aluminosilicate microspheres—a quartz mineral.

The hardening process of the gypsum binder has been described by several theories to date. According to the classical theory of A. A. Baikov, the hardening of gypsum binders proceeds in 3 stages, where stage 1 consists in the dissolution of semi-aqueous gypsum: the solution is quickly saturated, and its supersaturation occurs. At stage 2, setting (colloidalization) occurs, the resulting products cannot dissolve in the saturated liquid phase and are released in the form of finely dispersed colloidal particles, bypassing dissolution. The mass loses plasticity, but does not acquire strength; there is no adhesion between the hydrated particles. Stage 3—this is directly the period of crystallization, here the recrystallization of colloidal particles into large crystals and the formation of an intergrowth occurs. The hardening process of the samples of modified gypsum stone described in the study does not differ significantly, and the characteristics of the stone are influenced mainly by the shape and size of the crystals formed in the presence of mineral additives.

The optimal composition of the composition according to the criteria of strength and average density is provided with the introduction of slaked lime in the amount of 10% and aluminosilicate microspheres in the amount of 6% by weight of the gypsum binder, respectively. The technological characteristics of the raw mixes of the proposed compositions and the performance characteristics of the modified stone allows us to provide a complex of chemical additives. It is also worth noting the expected economic effect due to the use of an aluminosilicate component in the composition [32,33].

## 5. Conclusions

An aluminosilicate microsphere with a carbon content of about 3% can be isolated from the ash and slag mixture by hydraulic removal. With the joint introduction of the aluminosilicate component and the alkaline additive, the structural, physical and mechanical properties of the modified composition are improved. At the age of 7 days of air hardening, samples of modified gypsum stone with a complex (lime and aluminosilicate microspheres additives) have the highest compressive strength. It has been established that the aluminosilicate component in the amount of 4% in combination with slaked lime in the amount of 10% makes it possible to more than double the strength of the modified stone. It is also worth noting the improvement of other characteristics that facilitate the work with the material: the evenness of the resulting surface, the absence of sagging in the hardened layer and air bubbles, which is achieved due to the smooth and spherical surface of the aluminosilicate component. Thus, the studies have confirmed the effectiveness of using an aluminosilicate product for the enrichment of ash and slag mixtures as a component of compositions based on low-grade gypsum binder. Prescription compositions of compositions with chemical additives have been developed. They make it possible to provide the specified performance properties of compositions using aluminosilicate microspheres. This makes it possible to use them in the production of self-leveling floors, plastering and puttying works. The use of aluminosilicate microspheres, which are enriched with energy waste products, as part of a modified gypsum binder, has a positive effect on creating comfortable conditions for human living and on the preservation of the natural environment, that is, by reducing landfills from man-made waste. The use of waste makes it possible to reduce the percentage of areas allocated for landfills and the burial of man-made waste. Reducing the area under landfills is an important step towards solving such environmental problems as soil and water pollution, reducing the habitat of many animals.

## Figures and Tables

**Table 1:** Chemical composition of the aluminosilicate microsphere.

SiO_2_	MgO	CaO	Fe_2_O_3_	K_2_O	Al_2_O_3_	MnO	TiO_2_	P_2_O_5_	SiO_3_	C	Na_2_O
57.38	1.68	4.42	4.83	2.21	24.9	0.11	0.95	0.42	0.11	3.36	0.63

**Table 2:** Mineralogical composition of the control (1) and modified compositions (2) of gypsum stone.

N°	Gypsum	Anhydrite	Calcite	Dolomite	Ettringite	Quartz	Nacrite
1	72.1	0.5	15.9	4.7	5.3	1.5	-
2	64.5	0.8	12.9	3.6	13.4	2.6	2.2

**Table 3:** The chemical composition of the modified gypsum stone in weight and atomic %.

Spectrum	O	Mg	Al	Si	S	K	Ca	Fe	Result
in weight %									
Spectrum 1	59.26	2.67	8.39	19.78	1.61	1.88	4.01	2.40	100.00
Spectrum 2	62.67		0.19	0.66	15.90		20.58		100.00
Spectrum 3	57.81	0.32	0.26	1.02	16.81		23.77		100.00
Max	62.67	2.67	8.39	19.78	16.81	1.88	23.77	2.40	
Min	57.81	0.32	0.19	0.66	1.61	1.88	4.01	2.40	
in atomic %									
Spectrum 1	73.05	2.16	6.13	13.89	0.99	0.95	1.97	0.85	
Spectrum 2	79.02		0.15	0.47	10.00		10.36		
Spectrum 3	75.43	0.28	0.20	0.76	10.95		12.38		
Max	79.02	2.16	6.13	13.89	10.95	0.95	12.38	0.85	
Min	73.05	0.28	0.15	0.47	0.99	0.95	1.97	0.85	

**Table 4:** Prescription formulations.

Components, Mass. %	Composition 1	Composition 2
Gypsum binder G-4 grade	85.48	-
Gypsum binder G-5 grade	-	85.48
Lime slaked	7.8	7.8
Aluminosilicate microsphere	6.3	6.3
Hyperplasticizer	0.13	0.13
Powder defoamer	0.13	0.13
Wine acid	0.05	0.05
Water retention additive	0.05	0.05
Shrinkage compensator	0.06	0.06

## Data Availability

Not applicable.
